# The Role of Household Social Support and Undermining in Dietary Change

**DOI:** 10.1007/s12529-024-10327-w

**Published:** 2024-10-22

**Authors:** Marny M. Ehmann, Charlotte J. Hagerman, Brandy-Joe Milliron, Meghan L. Butryn

**Affiliations:** 1 Center for Weight, Eating, and Lifestyle Science, Department of Psychological and Brain Sciences, Drexel University, Philadelphia, PA 19104 USA; 2 Department of Nutrition Sciences, College of Nursing and Health Professions, Drexel University, Philadelphia, PA 19104 USA

**Keywords:** Dietary intake, Social support, Social undermining, Household, Dietary intervention

## Abstract

**Background:**

US adults find it challenging to meet disease prevention dietary recommendations and may participate in interventions to improve dietary quality. Social influences outside of the intervention, including level of social support and undermining of healthy eating in the home, may affect an individual’s dietary intake. This secondary analysis examined (1) changes in household social support and undermining of healthy eating across a dietary intervention with household member participation and (2) the relationship between changes in social influences and dietary intake.

**Method:**

Adults (*N* = 62) with low adherence to cancer prevention dietary recommendations recruited from the Philadelphia area participated in a 20-week dietary intervention focused on psychoeducation about NCI dietary recommendations and skills for behavior change. Half of the participants were also randomized to have an adult household member participate in some intervention contacts with them. Participants completed measures of social support and undermining of healthy eating and dietary intake at baseline and post-treatment (20 weeks).

**Results:**

Fifty-two participants had available data for baseline and post-treatment (i.e., completers). Household social support of healthy eating increased more among participants randomized to have household involvement in the intervention with a medium effect (*η*^2^ = .11). Fruit and vegetable intake significantly increased among participants with meaningful increases in household social support with a large effect (*η*^2^ = .37). There were no significant interaction effects of change in household undermining and time on change in dietary intake.

**Conclusion:**

Dietary interventions with a household support component show promise for improving household social support and may impact magnitude of dietary change.

## Introduction

Quality of dietary intake influences the risk of numerous health conditions [1, 2], yet most adults find it challenging to follow dietary guidelines for disease prevention [3]. For example, among adults in the USA, 57% of daily energy intake comes from ultra-processed foods [4] and only 10% of adults eat the daily recommended number of fruits and vegetables [5]. To improve dietary quality, adults may participate in interventions that enhance nutritional knowledge and skills for behavior change [6, 7]. However, according to the social cognitive theory [8], social influences outside of these interventions may affect one’s ability to make dietary changes [9, 10] by modulating self-regulatory processes, such as self-efficacy, and establishing norms for eating behavior [11–13]. Household social influences may be particularly consequential, given that roughly 70% of US adults live with at least one other adult and the majority of food consumption occurs in the home [14, 15].

Social influences in the household include both support and undermining of healthy eating. Social support of healthy eating can be operationalized as household members encouraging reduction in unhealthy food intake, discussing healthy eating habits, giving reminders to refrain from unhealthy foods, and giving compliments for following a healthy eating plan [16]. More frequent social support of healthy eating from family has been cross-sectionally related to lower levels of fat intake [17–19] and higher fruit and vegetable intake [17, 18, 20, 21]. In comparison to social support, social undermining has been given less attention, despite it being distinct from a *lack* of or *low* social support and theoretically associated with reduced self-efficacy for dietary change [22, 23]. Examples of social undermining of healthy eating by a household member include modeling unhealthy eating, refusal to eat healthy foods, bringing unhealthy foods into the home and/or offering unhealthy foods, and reacting critically to an individual’s attempts at increasing healthy food intake [16]. Previous cross-sectional research has found that frequent social undermining is associated with reduced confidence in controlling eating, perceived social pressure to eat [24], and poorer dietary quality among college students [25], gay couples [26], and those with diabetes [27].

Given the potential impact of household social support and undermining on dietary quality, it is important to examine the role of these influences in interventions that target dietary change. Only a few studies have examined how social support and undermining of healthy eating change over time in dietary interventions, and findings are mixed [28–31]. Furthermore, no previous studies to our knowledge have examined whether changes in household social support and undermining are related to changes in dietary intake over time among adults. Some researchers have begun to include household members in components of dietary interventions in an attempt to harness the influence of the household environment for change [32]. For example, studies have shown that parental engagement in components of dietary interventions targeting youth improves dietary quality among children and adolescents [33–37]. To extend this research to adults, our team examined whether having an adult household member join the index participant in select intervention components of a dietary intervention would enhance dietary change [38]. Dietary interventions with adult household member involvement have the potential to enhance buy-in and motivation for household dietary change by providing household members with nutrition education, facilitating discussion about household food dynamics, teaching effective communication and problem-solving skills, and setting goals to address barriers to change. Results from this parent study showed that participants randomized to have household involvement in the intervention had significantly greater reductions in their ultra-processed food and red and processed meat intake from baseline to post-treatment (i.e., 20 weeks), compared to those without household involvement, suggesting that involving adult household members helps facilitate healthy dietary changes [39]. More information from this parent study is needed to understand whether household involvement influences changes in social support and undermining across the intervention and how these changes relate to dietary outcomes.

Therefore, the current study builds on the findings of the parent study by measuring changes in household social support and undermining during this intervention and examining their relationships to dietary intake. Aim 1 examined changes in household social support and undermining of healthy eating across time (baseline to 20 weeks) within the full sample and by household involvement condition. Aim 2 examined whether changes in social support and undermining were related to changes in dietary intake from baseline to post-treatment (20 weeks).

## Methods

### Participants

Data for this secondary analysis were collected from adults (i.e., “index participants”; *N* = 62, age ≥ 18 years) enrolled in a National Cancer Institute (NCI)-funded proof-of-concept randomized controlled trial (Clinical Trials Identifier: NCT04947150) testing the effects of a 20-week, multicomponent dietary intervention on changes in dietary quality [38]. Index participants were recruited from the Philadelphia area in two cohorts using mailings, social media postings, and community engagement via the Regional Liaison Office at Thomas Jefferson University’s Sidney Kimmel Cancer Center. Inclusion criteria for the parent study included age 18 or older, low baseline adherence to NCI dietary recommendations for cancer prevention at baseline (i.e., operationalized as a score of ≤ 2 out of 4 on the NCI scoring scale) [40], and living with at least one adult household member who was willing to participate in select intervention contacts with the participants. Participants also were required to have access to a smartphone and report shopping at grocery stores that could passively stream item-level data from the store loyalty card to a project portal (i.e., Walmart, Wegmans, Target, Shoprite). Exclusion criteria included a medical condition that would limit appropriateness or adherence with intervention, involvement in another lifestyle modification program, bariatric surgery history, and pregnancy or breastfeeding. The parent study was approved by the Institutional Review Board at the supporting institution via expedited review and all index participants and enrolled household members provided written informed consent for participation.

### Procedures

Detailed study design and intervention methods for the parent study have been previously published elsewhere [38, 39]. Briefly, the parent study tested a 20-week intervention designed to improve dietary quality consistent with the guidelines for dietary cancer prevention by the World Cancer Research Fund/American Institute for Cancer Research: (1) eat a diet rich in whole grains, vegetables, and fruit; (2) limit consumption of highly processed foods; (3) limit consumption of red and processed meat; and (4) eliminate consumption of sugar-sweetened beverages [1]. All index participants attended three workshops (90 min each; via Zoom videoconferencing) delivered by M.S. or Ph.D. level coaches with degrees in psychology, nutrition, or a related field and received a once weekly text message from the program. Workshops and text messages focused on providing education and teaching behavioral skills (e.g., goal-setting, meal planning) to improve adherence to the dietary recommendations for cancer prevention. Index participants also were randomized to receive additional intervention components, staggered throughout the 20 weeks, using a factorial design where four experimental conditions were ON or OFF for each participant. Index participants were independently randomized to ON vs OFF for each of the four experimental intervention components (i.e., the 4 experimental intervention components were not “bundled” together), yielding 16 different combinations of intervention components. These four experimental conditions included the following: (1) location-triggered messaging, in which the weekly study message was sent at the grocery store, as opposed to a fixed schedule, to increase salience of dietary goals in the moment; (2) benefits of change, in which participants reflected on the personally meaningful short- and long-term benefits of change in one additional workshop (60 min) and three additional phone calls (20 min); (3) coach monitoring, in which a coach (i.e., study staff member) provided personalized feedback on dietary changes via three additional phone calls (20 min) and additional text messages; and (4) household member involvement, in which an adult household member joined index participants in one additional workshop (60 min) and three additional coaching calls (20 min) to improve household support for dietary changes.

For those randomized to have household member involvement ON, the role of the household member was to serve as a support person for the index participant. Health behavior change determinants including support regulation, tailored support, and disclosure were addressed by the dyads targeted at the behavior change in the index participant, per the compendium of dyadic interventions [41]. Household members received education about the NCI dietary recommendations for cancer prevention and the impact of the home food environment on dietary change, and household dyads received dedicated time to discuss barriers to household changes, household food dynamics, and ways to provide effective supportive communication for dietary change. As such, most intervention tasks were joint, performed by the couple together and targeting the index participant, per the dyadic intervention continuum [41]. Household members also received a weekly text message, which is a cross-over task with the directive given to the household member alone to support the index participant.

Aspects of the household intervention that targeted social support were discussion about how the household member facilitates healthy eating, collaborative goal setting, and supportive communication skills practice. Aspects of the intervention that targeted social undermining were discussion of how the household member makes healthy eating challenging and problem-solving when index participant felt unsupported. Household members had no intervention involvement when index participants were not randomized to household support condition.

### Measures

#### Participant Characteristics

Participants self-reported age, sex, height, weight, race, ethnicity, education, employment status, income, and household composition at baseline.

#### Household Social Support and Undermining of Healthy Eating

Household social support and undermining of healthy eating were measured using the Sallis Social Support for Diet questionnaire [16] at baseline and post-treatment (20 weeks). The Sallis scale has been widely used in previous research to measure support for health behavior engagement and has shown adequate validity and internal consistency [24]. This 10-item measure was used to assess the perceived frequency of support (5 items; e.g., “complimented me on changing my eating habits”) and undermining (5 items; e.g., “ate unhealthy foods in front of me”) of healthy eating from adult household members. Participants rated each item on a scale from 1 (none) to 5 (very often), with an additional option to indicate not applicable. Items scored as not applicable were recoded to 1 s (none). Items on the support and undermining subscales were added together to comprise a total score for each subscale. Higher scores on each subscale indicated higher household social support and undermining of healthy eating. One adaptation was made to the question stem of the scale to assess support and undermining from adult household member (instead of family more globally): “Please rate how often your *adult household member(s)* have said or done what is described.”

#### Dietary Intake

Outcome variables included daily intake of fruits and vegetables (cups/day), added sugars (grams/day), saturated fats (grams/day), and sodium (mg/day) from processed foods, red (oz/week) and processed (grams/week) meat, and sugar-sweetened beverages (oz/day). Dietary intake was measured at baseline and post-treatment (20 weeks) using the Automated Self-Administered 24-h Dietary Recall (ASA-24) or the Diet History Questionnaire (DHQ-III). Participants in cohort 1 of recruitment completed 3 days of food recall (Saturday, Tuesday, and another weekday) using the ASA-24, a measure with a well-validated automated multiple pass method which has been shown to be as accurate as nutritionist-administered 24-h food recall [42, 43]. Because of the pilot nature of the parent study, the research team decided to measure dietary intake using the DHQ-III for participants in cohort 2 after cohort 1 participants provided feedback that the ASA-24 was excessively burdensome. The DHQ-III is a food frequency questionnaire developed by the National Cancer Institute based on national dietary recall data from the National Health and Nutrition Examination Surveys [44]. Both methods provide validated item-level nutritional data by measuring the frequency of intake of all foods and drinks consumed, and each captures the relevant dietary outcomes for the present study in a comparable fashion.

Fruit and vegetable intake included daily intake of all fruit (intact whole or cut, not including fruit juices) and all vegetables except starchy vegetables. Highly processed foods were flagged via the NOVA classification system and included salty snacks, frozen and prepared meals, baked goods, dessert, fried potatoes, candy, packaged bread and buns, and refined grains [45]. Red meat included beef, veal, pork, lamb, or game, and processed meat included frankfurters, sausages, corned beef, and luncheon meat made from beef, pork, or poultry. Sugar-sweetened beverages included non-diet sodas, non-diet fruit drinks, energy drinks, and sugary coffee drinks. Daily intake of fruits and vegetables, added sugars, sodium, and saturated fats from processed foods, red and processed meat, and sugar-sweetened beverages was calculated using pre-established ASA-24 (Food and Nutrient Database food code) and DHQ-III (NCI nutrient database) methods [44, 46].

### Data Analysis

Data were analyzed using SPSS 28.0 [47] and the significance level was set to 0.05. Study sample characteristics were measured using descriptive statistics (means, SDs, frequencies). Of the participants who enrolled in the study (*N* = 62), ten did not provide post-treatment data. Non-completers did not significantly differ from completers in baseline BMI, social support, undermining, red and processed meat intake, sugar-sweetened beverage intake, sodium intake, added sugar intake, or saturated fat intake (*p*s > 0.08), but were younger (*p* = 0.03) and had less fruit and vegetable intake at baseline (*p* = 0.03). All analyses were conducted among completers (*n* = 52) and among the full sample using the single imputation method baseline observation carried forward, in which the baseline value was imputed for the post-treatment missing value in non-completers (*n* = 10). The pattern of results did not differ when analyzing the completers only and the full sample; therefore, completer analyses are presented in the “Results” section.

To test **Aim 1**, repeated measures ANCOVAs examined whether household social support and undermining changed across time (i.e., from baseline to post-treatment). To assess whether this change differed by household member involvement, models included the interaction effects of household member condition and time (0 = OFF, 1 = ON). We reported simple effects within the interactions, examining change over time when household member condition is OFF and ON, respectively.

To test **Aim 2**, repeated-measures ANCOVAs examined whether change in dietary intake from pre- to post-treatment (20 weeks) was moderated by change in household social support or undermining over time. Change in household social support and undermining was calculated as a change score from baseline to post-treatment, with positive scores indicating increases in and negative scores indicating reductions in support or undermining. This change score was categorized into three groups for analysis: (1) those who had a meaningful increase in support or undermining (i.e., score increased by ≥ 3 points from baseline to post-treatment); (2) those with no/minimal change in support or undermining (post-treatment score was within 2 points of baseline score); and (3) those who had a meaningful reduction in support or undermining (i.e., score decreased by ≥ 3 points from baseline to post-treatment). Transforming continuous social support and undermining change scores into this categorical grouping variable was done to improve interpretability of results. Aim 2 models were run with change in social support or undermining as continuous variables, and the pattern of results did not differ from models in which the categorical variables were included. As such, the categorical approach was retained.

Models included the interaction effects of change in social influences (undermining or support) as a categorical grouping variable and time. To probe significant interactions, we examined the predicted change in dietary intake over time among the three levels of the grouping variable described above: meaningful increase, no/minimal change, and meaningful reduction in support or undermining. Each experimental condition variable was coded as a categorical variable with 0 = OFF or 1 = ON, and all Aim 2 models controlled for experimental conditions and baseline social support or baseline social undermining. Research suggests that reported dietary intake may differ by type of dietary assessment, with more pronounced differences for total fruits, greens and beans, fatty acids, refined grains, sodium, and saturated fats [48]. In the present study, there were no significant differences in mean intake of fruits and vegetables, added sugars, sugar-sweetened beverages, and processed meats between participants who completed the ASA-24 and those who completed the DHQ-III at both time points (*p*s > 0.07). However, there were significant mean differences in sodium, saturated fat, and red meat intake daily (*p*s < 0.05), with lower reported intake by participants who completed the DHQ-III. As such, all Aim 2 models also controlled for type of dietary measure completed (1 = ASA-24, 2 = DHQ-III). Outliers with a standardized residual of ≥|3| were excluded in analyses for hypotheses tests. Age, BMI, and race (White and non-White), measured at baseline, were tested as covariates in Aim 1 and 2 models, but were not significantly related to outcomes (*p*s > 0.05), and were not included in models.

## Results

### Participant Characteristics

Baseline index participant characteristics are presented in Table 1. There were no statistically significant differences in household social support, undermining, or dietary intake among experimental conditions at baseline (*p*s < 0.05).

**Table 1:** Baseline participant characteristics (*N* = 62)

Age (*M* ± SD)	47.2 ± 13.5
BMI (*M* ± SD)	32.1 (8.0)
Sex, *n* (%)	
Male	5 (8.1%)
Female	57 (91.9%)
Race, *n* (%)	
Non-White	29 (4.8%)
White	33 (53.2%)
Ethnicity,* n* (%)	
Hispanic	6 (9.7%)
Non-Hispanic	56 (90.3%)
Education, *n* (%)	
Completed high school	3 (4.8%)
Completed some college	12 (19.4%)
Graduated from college	29 (46.8%)
Postgraduate or professional degree	18 (29%)
Employment status, *n* (%)	
Full-time	34 (54.8%)
Part-time	14 (22.6%)
Occasional	2 (3.2%)
Disability/SSI	2 (3.2%)
No income	10 (16.1%)
Income, *n* (%)	
< $60,000 per year	17 (27.4%)
≥ $60,000 per year	36 (58.1%)
Unknown/not reported	9 (14.5%)
Number of individuals in the home (excluding index participants), *n* (%)	
One other person	24 (38.7%)
Two other people	15 (24.2%)
Three other people	16 (25.8%)
Four or more other people	7 (11.2%)
Number of adults in the home (including index participants), *n* (%)	
Two adults	44 (71%)
Three adults	14 (22.5%)
Four adults	4 (6.5%)
Number of minor children in the home, *n* (%)	
Zero children	32 (51.6%)
One child	13 (21%)
Two children	11 (17.7%)
Three children	4 (6.5%)
Four children	2 (3.2%)

### Aim 1: Change in Social Influences Across Time

Table 2 shows change over time in social support and undermining for the completer sample and by household member involvement. Repeated measures ANCOVAs showed no significant change in household social undermining of healthy eating (*F*(1,47) = 2.07, *p* = 0.16, *η*^2^ = 0.04) or household social support of healthy eating (*F*(1,47) = 0.87, *p* = 0.36, *η*^2^ = 0.02) from baseline to post-treatment across all participants. The interaction between time and household involvement on household social undermining was not significant (*F*(1,47) = 0.68, *p* = 0.41, *η*^2^ = 0.01). However, there was a significant interaction between time and household member involvement on household social support of healthy eating, with a medium effect size (*F*(1,47) = 5.68, *p* = 0.02, *η*^2^ = 0.11). Household social support of healthy eating significantly increased among participants with household involvement in the intervention (*p* = 0.02) but not among those without household involvement in the intervention (*p* = 0.31). The pattern of results remained the same when examining the full sample with baseline social support and social undermining values imputed at post-treatment for those who did not complete post-treatment questionnaires (*n* = 10).

**Table 2:** Change in social influences from baseline to post-treatment for the full sample and separated by randomization to household involvement

	*N*	Household social support				Household social undermining			
		Baseline	Post-treatment	*M* change	*p* value	Baseline	Post-treatment	*M* change	*p* value
All participants	52	13.3	13.9	+ 0.6	0.36	13.5	12.4	− 1.1	0.16
Household OFF	25	14.2	13.2	− 1.0	0.31	13.4	12.9	− 0.50	0.70
Household ON	27	**12.4**	**14.7**	** + 2.3**	**0.02**	13.6	11.9	− 1.7	0.11

Bolded items = *p* < 0.05

Possible social undermining and support scores range from 5 to 25. Higher scores indicate higher support or undermining

Models controlled for other experimental conditions (location triggered messaging, benefits of change, and coach monitoring)

### Aim 2: Relationship Between Change in Social Influences and Dietary Intake

**Fig. 1:**
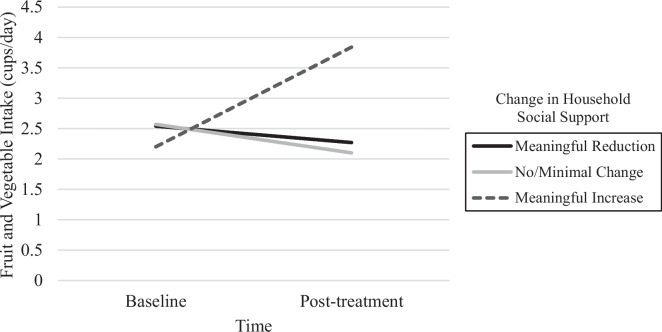
Change in daily fruit and vegetable intake (cups) from baseline to post-treatment (20 weeks) by change in household support of healthy eating. *Note:* Meaningful reduction group = post-treatment score decreased by ≥ 3 points from baseline (range =  − 10 to − 3 points); no/minimal change group = post-treatment score within 2 points of baseline score; meaningful increase group = post-treatment score increased by ≥ 3 points from baseline (range =  + 3 to 16 points)

Repeated measures ANCOVAs showed that there was a significant interaction between time and change in household social support of healthy eating for daily intake of fruit and vegetables, with a large effect size (*F*(1,42) = 12.57, *p* =  < 0.001, *η*^2^ = 0.37). Post hoc analyses examined change in fruit and vegetable intake over time among three groups: those with a meaningful reduction in household social support from baseline to post-treatment (*n* = 11; *M* change = 5.6 ± 1.9-point decrease from baseline), those with no/minimal changes in household social support from baseline to post-treatment (*n* = 23; *M* change = 0.4 ± 1.4-point change from baseline), and those with meaningful increases in household social support from baseline to post-treatment (*n* = 17; *M* change = 6.1 ± 3.3-point increase from baseline). Post hoc analyses showed that there was a significant increase in fruit and vegetable intake from baseline (*M* = 2.2 cups/day) to post-treatment (*M* = 3.8 cups/day) among those who reported a meaningful increase in household social support of healthy eating (*p* < 0.001). Those who reported no/minimal change or a meaningful reduction in household social support had non-significant decreases in fruit and vegetable intake from baseline to post-treatment (*ps* > 0.08; see Fig. 1). There were no significant interaction effects of change in household social support and time on change in processed food, sugar-sweetened beverage, or red and processed meat intake from baseline to post-treatment. There were no significant interaction effects of change in household social undermining of healthy eating and time on change in dietary variables (*p*s > 0.05). The pattern of results remained the same when using the full sample with baseline observation carried forward imputation for those who did not complete post-treatment questionnaires (*n* = 10).

## Discussion

Dietary quality has been shown to be related to the risk of developing various health conditions, and research has reported that social support and social undermining of healthy eating are primary facilitators and barriers of healthy dietary intake, respectively [49–52]. Given these findings, various dietary and weight control behavior change interventions have begun to include household members or members of the social network (e.g., romantic partner) to participate alongside the index participant in the intervention [53–55]. It was previously shown that participants who were randomized to involve an adult household member in some intervention contacts had significantly greater reductions in their ultra-processed food and red and processed meat intake from baseline to post-treatment than those randomized to receive the intervention without a household member. This secondary data analysis expanded on these results by examining how household social support and undermining of healthy eating changed across the 20-week dietary intervention and investigated whether these changes were related to changes in dietary intake at post-treatment.

Among the entire sample, household social support and undermining of healthy eating did not significantly change over time. Previous studies have also found that multicomponent dietary or weight control interventions do not significantly improve social support [28, 29, 56] or reduce social undermining of healthy eating [57]. However, the interaction between household involvement and time on household social support of healthy eating was significant, such that participants who had a household member involved in the intervention reported an average of 2.3-point increase in household social support of healthy eating during the intervention, as compared to a 1-point decrease among those without household support in the intervention. Sorkin and colleagues reported a similar result in a dyadic lifestyle modification program, such that enrolled pairs of mothers and adult daughters reported significantly greater social support post-intervention [58]. This suggests that providing psychoeducation to adult household members about cancer prevention dietary recommendations and encouraging them to collaborate with the index participant on household food environment, meal planning, and grocery shopping changes may be an effective way to increase support of healthy eating in the household. It is unclear from the current study which specific components of the household member contacts significantly improved perceived household support of healthy eating; therefore, future research should test what core interventions are necessary to produce these changes.

In comparison to household support, the interaction between household involvement and time on household social undermining of healthy eating was not significant, suggesting that the household engagement component may not target aspects of an undermining environment as much as facilitate support and encouragement. This aligns with previous research from Frerichs et al. [31] that found that the inclusion of family members and education on social support in a dietary intervention did not reduce family undermining of healthy eating. This may be because social undermining has received little research attention compared to social support, despite it being conceptually distinct from supportive social processes [22]. Interventions may readily focus on ways to increase social support for health behavior changes, as opposed to discussing or providing strategies for managing statements and behaviors that discourage healthy dietary intake. For example, the intervention used in the current study provided dedicated time to discuss how the household member facilitates healthy eating, collaborative goal setting to improve index participant diet, and skills practice in supportive communication. Some aspects of social undermining were addressed, such as how the index participant makes healthy eating more challenging and ways that the index participant feels unsupported. However, aspects of social undermining, such as criticism, complaint, minimization, and forms of interference of healthy eating, may not have been as emphasized. Future research should further investigate which factors may be most salient to undermining and introduce more targeted education and intervention for this type of social interaction.

The current study was also the first to the authors’ knowledge to examine the relationship between changes in household social support and undermining and dietary changes in a sample of adults following a dietary intervention. Results showed those who reported a meaningful increase in household support of healthy eating throughout the intervention, compared to those whose household support did not change or decreased, significantly increased their fruit and vegetable intake from baseline to post-treatment. Rotman and colleagues [57] found that greater increases in family support of healthy eating from pre- to post-treatment were associated with greater increases in vegetable intake in children. It could be that those who experienced an increase in encouraging behaviors and communication around healthy eating in the home were more motivated to and had greater buy-in for following recommendations for fruit and vegetable intake.

However, contrary to hypotheses, there were no significant interaction effects of time and changes in household undermining on dietary intake. This study is consistent with a previous study that found that changes in social undermining were not significantly related to changes in dietary intake among children following a family-based dietary intervention [57]. As previously mentioned, it could be that intervention targets focused more on building up social support than addressing social undermining or that the available measures to assess social undermining do not adequately capture the variety in types suggested by qualitative literature [59]. It could also be that the positive aspects of social support buffered against social undermining, attenuating any effects it might have had on dietary intake. Some studies have found direct associations between social undermining of healthy eating and poorer diet in individuals with existing diabetes whose diets are specialized and complex [27]. Furthermore, Novak and colleagues [26] found that romantic partner undermining was related to poorer dietary intake through couple food-related disagreements and heightened depressive symptoms among older, gay couples. Future research should continue to examine the relationship between social undermining and dietary intake, and test intervention components targeted toward social undermining.

There are several limitations to the current study. The study sample size was small, which limited the power to assess outcomes with robust multilevel models, conduct mediational analyses, or examine three-way interactions. Furthermore, ANOVAs utilize listwise deletion, which includes data from participants who completed baseline and post-treatment assessments only (*n* = 52; i.e., completers). Analyses utilizing baseline observation carried forward to impute missing post-treatment values in non-completers (*n* = 10) did not significantly differ from analyses using completers only. More robust methods of handling missing data (i.e., multiple imputation) should be used in future studies to replicate findings. Due to the small sample, the study is also underpowered to detect small effects, and there may be relationships that did not emerge as statistically significant in the present study. Furthermore, women, adults with college education, and adults with overweight/obesity were all overrepresented in the sample, limiting the ability to assess gender differences in outcomes and limiting generalizability to other populations. Additionally, it is unknown how well maintained these changes in social influences were, given that the study did not include a follow-up period. Future research should examine household support and undermining among larger, more diverse populations of adults attempting to improve dietary quality over a longer period. Finally, the study used two different self-report dietary assessments to measure dietary intake in cohort 1 and cohort 2. There could be differences in reported intake by the type of dietary assessment used; however, study analyses included type of dietary measure in models to control for this effect and both assessments provide comparable dietary variables of interest in the study. There also is a possibility that participants unintentionally under- or over-reported aspects of their intake using self-report measures, which is a common challenge in research on dietary intake. When tested against biomarkers, self-reported protein, energy, potassium, and sodium are underreported by 15–17% using the ASA24 and 29–34% using the DHQ-II [60]. However, in general, the ASA-24 has been shown to capture approximately 80% of foods and drinks consumed [42].

Despite these limitations, the study has several strengths. The current study investigated both household social support and undermining in a lifestyle intervention. Discouraging social interactions are generally not measured in behavioral interventions, despite theory that points to the saliency of negative social interactions in influencing behavioral changes. Furthermore, the current study examined social influences in conditions in which participants were randomized to receive additional support from household members, allowing for a more robust comparison of the impact of supportive content on household support and undermining. Although this was not tested, household members may also have personally benefitted from involvement in the intervention (i.e., ripple effect), which has been shown in previous studies [61].

Participants in a behavioral intervention for dietary cancer prevention reported significant increases in household social support of healthy eating, but no changes in household undermining of healthy eating, when an adult household member participated alongside the index participant in select intervention contacts. Dietary interventions with a household support component show promise for improving household support of healthy eating, but current iterations may not provide adequate assistance in managing critical remarks or behaviors that undermine healthy dietary intake. Improvements in household support were also related to improvements in fruit and vegetable intake across the intervention, highlighting the importance of household social influences when designing dietary interventions. There is more work to be done to determine which specific support components may improve support of healthy eating and to identify the missing interventions that target social undermining.

## Data Availability

The data generated during this study are available from the corresponding author upon reasonable request.

## Declarations

### Welfare of Animals

This article does not contain any studies with animals performed by any of the authors.

## References

[1] Clinton SK, Giovannucci EL, Hursting SD. The world cancer research fund/american institute for cancer research third expert report on diet, nutrition, physical activity, and cancer: impact and future directions. J Nutr. 2020;150(4):663–71. 10.1093/jn/nxz268. 31758189 10.1093/jn/nxz268 PMC7317613

[2] Lagström H, Stenholm S, Akbaraly T, et al. Diet quality as a predictor of cardiometabolic disease–free life expectancy: the Whitehall II cohort study. Am J Clin Nutr. 2020;111(4):787–94. 31927573 10.1093/ajcn/nqz329 PMC7138656

[3] Liu J, Micha R, Li Y, Mozaffarian D. Trends in food sources and diet quality among US children and adults, 2003–2018. JAMA Network Open. 2021;4(4):e215262-e. 10.1001/jamanetworkopen.2021.5262 PMC8042524 33844000

[4] Juul F, Parekh N, Martinez-Steele E, Monteiro CA, Chang VW. Ultra-processed food consumption among US adults from 2001 to 2018. Am J Clin Nutr. 2021;115(1):211–21. 10.1093/ajcn/nqab305. 10.1093/ajcn/nqab305 34647997

[5] Lee SH. Adults meeting fruit and vegetable intake recommendations—United States, 2019. MMWR Morbid Mortal Weekly Rep. 2022;71:1–9. 10.15585/mmwr.mm7101a1 PMC8735562 34990439

[6] Browne S, Minozzi S, Bellisario C, Sweeney MR, Susta D. Effectiveness of interventions aimed at improving dietary behaviours among people at higher risk of or with chronic non-communicable diseases: an overview of systematic reviews. Eur J Clin Nutr. 2019;73(1):9–23. 30353122 10.1038/s41430-018-0327-3

[7] Young C, Campolonghi S, Ponsonby S, et al. Supporting engagement, adherence, and behavior change in online dietary interventions. J Nutr Educ Behav. 2019;51(6):719–39. 10.1016/j.jneb.2019.03.006. 31036500 10.1016/j.jneb.2019.03.006

[8] Bandura A. Social cognitive theory: an agentic perspective. Annu Rev Psychol. 2001;52(1):1–26. 10.1146/annurev.psych.52.1.1. 11148297 10.1146/annurev.psych.52.1.1

[9] Enriquez JP, Archila-Godinez JC. Social and cultural influences on food choices: A review. Crit Rev Food Sci Nutr. 2022;62(13):3698–704. 10.1080/10408398.2020.1870434. 33427479 10.1080/10408398.2020.1870434

[10] Vadiveloo MK, Sotos-Prieto M, Parker HW, Yao Q, Thorndike AN. Contributions of food environments to dietary quality and cardiovascular disease risk. Curr Atheroscler Rep. 2021;23(4):14. 10.1007/s11883-021-00912-9. 33594516 10.1007/s11883-021-00912-9

[11] Ball K, Jeffery RW, Abbott G, McNaughton SA, Crawford D. Is healthy behavior contagious: associations of social norms with physical activity and healthy eating. Int J Behav Nutr Phys Act. 2010;7(1):86. 10.1186/1479-5868-7-86. 21138550 10.1186/1479-5868-7-86 PMC3018448

[12] Cruwys T, Bevelander KE, Hermans RCJ. Social modeling of eating: A review of when and why social influence affects food intake and choice. Appetite. 2015;86:3–18. 10.1016/j.appet.2014.08.035. 25174571 10.1016/j.appet.2014.08.035

[13] Higgs S, Liu J, Collins E, Thomas J. Using social norms to encourage healthier eating. Nutr Bull. 2019;44(1):43–52.

[14] America’s Families and Living Arrangements: 2022. 2022. https://www.census.gov/data/tables/2022/demo/families/cps-2022.html. Accessed 17 Aug 2024.

[15] Lin B-H, Guthrie J. Nutritional quality of food prepared at home and away from home, 1977–2008. EIB-105. U.S. Department of Agriculture, Economic Research Service. 2012. https://www.ers.usda.gov/webdocs/publications/43698/34513_eib-105.pdf?v=0. Accessed 21 Jul 2024.

[16] Sallis JF, Grossman RM, Pinski RB, Patterson TL, Nader PR. The development of scales to measure social support for diet and exercise behaviors. Prev Med. 1987;16(6):825–36. 3432232 10.1016/0091-7435(87)90022-3

[17] Anderson ES, Winett RA, Wojcik JR. Self-regulation, self-efficacy, outcome expectations, and social support: social cognitive theory and nutrition behavior. Ann Behav Med. 2007;34(3):304–12. 10.1007/bf02874555. 18020940 10.1007/BF02874555

[18] Anderson-Bill ES, Winett RA, Wojcik JR. Social Cognitive Determinants of Nutrition and Physical Activity Among Web-Health Users Enrolling in an Online Intervention: The Influence of Social Support, Self-Efficacy, Outcome Expectations, and Self-Regulation. J Med Internet Res. 2011;13(1):e28. 10.2196/jmir.1551. 21441100 10.2196/jmir.1551 PMC3221350

[19] Karfopoulou E, Anastasiou CA, Avgeraki E, Kosmidis MH, Yannakoulia M. The role of social support in weight loss maintenance: results from the MedWeight study. J Behav Med. 2016;39(3):511–8. 10.1007/s10865-016-9717-y. 26801339 10.1007/s10865-016-9717-y

[20] Ball K, Crawford D, Mishra G. Socio-economic inequalities in women’s fruit and vegetable intakes: a multilevel study of individual, social and environmental mediators. Public Health Nutr. 2006;9(5):623–30. 10.1079/phn2005897. 16923294 10.1079/phn2005897

[21] Dulin A, Risica PM, Mello J, et al. Examining neighborhood and interpersonal norms and social support on fruit and vegetable intake in low-income communities. BMC Public Health. 2018;18(1):455. 10.1186/s12889-018-5356-2. 29621989 10.1186/s12889-018-5356-2 PMC5887203

[22] Ogden J, Quirke-McFarlane S. Sabotage, collusion, and being a feeder: towards a new model of negative social support and its impact on weight management. Curr Obes Rep. 2023;12(2):183–90. 10.1007/s13679-023-00504-5. 37280423 10.1007/s13679-023-00504-5 PMC10250496

[23] Hardcastle S, Hagger MS. “You Can’t Do It on Your Own”: Experiences of a motivational interviewing intervention on physical activity and dietary behaviour. Psychol Sport Exerc. 2011;12(3):314–23. 10.1016/j.psychsport.2011.01.001.

[24] Rieger E, Sellbom M, Murray K, Caterson I. Measuring social support for healthy eating and physical activity in obesity. Br J Health Psychol. 2018;23(4):1021–39. 30054957 10.1111/bjhp.12336

[25] Alshehri M, Kruse-Diehr AJ, McDaniel JT, Partridge J, Null DB. Impact of social support on the dietary behaviors of international college students in the United States. J Am Coll Health. 2021;1–9. 10.1080/07448481.2021.1970565. 10.1080/07448481.2021.1970565 34449292

[26] Novak JR, Wilson SJ, Gast J, Miyairi M, Peak T. Associations between partner’s diet undermining and poor diet in mixed-weight, older gay married couples: a dyadic mediation model. Psychol Health. 2021;36(10):1147–64. 10.1080/08870446.2020.1836179. 33090040 10.1080/08870446.2020.1836179 PMC12178781

[27] Henry SL, Rook KS, Stephens MA, Franks MM. Spousal undermining of older diabetic patients’ disease management. J Health Psychol. 2013;18(12):1550–61. 10.1177/1359105312465913. 23325381 10.1177/1359105312465913 PMC4506743

[28] Uribe ALM, Demment M, Graham ML, et al. Improvements in dietary intake, behaviors, and psychosocial measures in a community-randomized cardiovascular disease risk reduction intervention: Strong Hearts, Healthy Communities 2.0. Am J Clin Nutr. 2023;118(5):1055–66. 37717638 10.1016/j.ajcnut.2023.09.003 PMC10636233

[29] Cyriac J, Jenkins S, Patten CA, et al. Improvements in diet and physical activity–related psychosocial factors among African Americans using a mobile health lifestyle intervention to promote cardiovascular health: The FAITH!(fostering African American improvement in Total health) app pilot study. JMIR Mhealth Uhealth. 2021;9(11):e28024. 34766917 10.2196/28024 PMC8663698

[30] Aschbrenner KA, Mueser KT, Naslund JA, et al. Facilitating partner support for lifestyle change among adults with serious mental illness: a feasibility pilot study. Community Ment Health J. 2017;53:394–404. 28176207 10.1007/s10597-017-0100-4 PMC5510867

[31] Frerichs L, Bess K, Young TL, et al. A cluster randomized trial of a community-based intervention among African-American adults: effects on dietary and physical activity outcomes. Prev Sci. 2020;21:344–54. 31925605 10.1007/s11121-019-01067-5 PMC7058497

[32] Ho Y-CL, Mahirah D, Ho CZ-H, Thumboo J. The role of the family in health promotion: a scoping review of models and mechanisms. Health Prom Int. 2022;37(6):119. 10.1093/heapro/daac119 PMC9673498 36398941

[33] Varagiannis P, Magriplis E, Risvas G, et al. Effects of three different family-based interventions in overweight and obese children: the “4 your family” randomized controlled trial. Nutrients. 2021;13(2):341. 33498894 10.3390/nu13020341 PMC7911878

[34] Snuggs S, Houston-Price C, Harvey K. Healthy eating interventions delivered in the family home: A systematic review. Appetite. 2019;140:114–33. 31091432 10.1016/j.appet.2019.05.014

[35] Pearson N, Atkin AJ, Biddle SJ, Gorely T. A family-based intervention to increase fruit and vegetable consumption in adolescents: a pilot study. Public Health Nutr. 2010;13(6):876–85. 20196908 10.1017/S1368980010000121

[36] Wilson DK, Sweeney AM, Quattlebaum M, Loncar H, Kipp C, Brown A. The moderating effects of the Families Improving Together (FIT) for weight loss intervention and parenting factors on family mealtime in overweight and obese African American adolescents. Nutrients. 2021;13(6):1745. 34063799 10.3390/nu13061745 PMC8224069

[37] Quick V, Martin-Biggers J, Povis GA, Worobey J, Hongu N, Byrd-Bredbenner C. Long-term follow-up effects of the HomeStyles randomized controlled trial in families with preschool children on social cognitive theory constructs associated with physical activity cognitions and behaviors. Contemp Clin Trials. 2018;68:79–89. 29549006 10.1016/j.cct.2018.03.006

[38] Horgan OZ, Crane NT, Forman EM, et al. Optimizing an mHealth Intervention to Change Food Purchasing Behaviors for Cancer Prevention: Protocol for a Pilot Randomized Controlled Trial. JMIR research protocols. 2022;11(6):e39669. 35749216 10.2196/39669 PMC9270710

[39] Butryn ML, Hagerman CJ, Crane NT, et al. A proof-of-concept pilot test of a behavioral intervention to improve adherence to dietary recommendations for cancer prevention. Cancer Control. 2023;30:10732748231214122. 37950612 10.1177/10732748231214122 PMC10640808

[40] Turati F, Dalmartello M, Bravi F, et al. Adherence to the world cancer research fund/american institute for cancer research recommendations and the risk of breast cancer. Nutrients. 2020;12(3):607. 32110887 10.3390/nu12030607 PMC7146587

[41] Di Maio S, Villinger K, Knoll N, et al. Compendium of dyadic intervention techniques (DITs) to change health behaviours: a systematic review. Health Psychol Rev. 2024;18(3):538–73. 10.1080/17437199.2024.2307534. 10.1080/17437199.2024.2307534 38437798

[42] Kirkpatrick SI, Subar AF, Douglass D, et al. Performance of the Automated Self-Administered 24-hour Recall relative to a measure of true intakes and to an interviewer-administered 24-h recall. Am J Clin Nutr. 2014;100(1):233–40. 10.3945/ajcn.114.083238. 24787491 10.3945/ajcn.114.083238 PMC4144101

[43] Moshfegh AJ, Rhodes DG, Baer DJ, et al. The US Department of Agriculture Automated Multiple-Pass Method reduces bias in the collection of energy intakes. Am J Clin Nutr. 2008;88(2):324–32. 10.1093/ajcn/88.2.324. 18689367 10.1093/ajcn/88.2.324

[44] Diet History Questionnaire III (DHQ III). National Cancer Institute: Division of Cancer Control & Population Sciences. Epidemiology and Genomics Research Program Web site. https://epi.grants.cancer.gov/dhq3/. Accessed 21 Jul 2024.

[45] Monteiro CA, Cannon G, Levy RB, et al. Ultra-processed foods: what they are and how to identify them. Public Health Nutr. 2019;22(5):936–41. 10.1017/s1368980018003762. 30744710 10.1017/S1368980018003762 PMC10260459

[46] Food and Nutrient Database for Dietary Studies (FNDDS) U.S. Department of Agriculture. https://data.nal.usda.gov/dataset/food-and-nutrient-database-dietary-studies-fndds. Accessed 21 Jul 2024.

[47] IBM Corp. IBM SPSS Statistics for Macintosh, Version 28.0. Armonk, NY: IBM Corp. Released 2021.

[48] Subar AF, Potischman N, Dodd KW, et al. Performance and feasibility of recalls completed using the automated self-administered 24-hour dietary assessment tool in relation to other self-report tools and biomarkers in the interactive diet and activity tracking in AARP (IDATA) study. J Acad Nutr Diet. 2020;120(11):1805–20. 32819883 10.1016/j.jand.2020.06.015 PMC7606702

[49] Berge JM, Larson N, Bauer KW, Neumark-Sztainer D. Are parents of young children practicing healthy nutrition and physical activity behaviors? Pediatrics. 2011;127(5):881–7. 21482603 10.1542/peds.2010-3218 PMC3081185

[50] van der Put A, Ellwardt L. Employees’ healthy eating and physical activity: the role of colleague encouragement and behaviour. BMC Public Health. 2022;22(1):2004. 36319982 10.1186/s12889-022-14394-0 PMC9628058

[51] Mackert M, Stanforth D, Garcia AA. Undermining of nutrition and exercise decisions: experiencing negative social influence. Public Health Nurs. 2011;28(5):402–10. 10.1111/j.1525-1446.2011.00940.x. 22092423 10.1111/j.1525-1446.2011.00940.x

[52] Munt AE, Partridge SR, Allman-Farinelli M. The barriers and enablers of healthy eating among young adults: a missing piece of the obesity puzzle: A scoping review. Obes Rev. 2017;18(1):1–17. 10.1111/obr.12472. 27764897 10.1111/obr.12472

[53] Gorin AA, Powers TA, Gettens K, et al. A randomized controlled trial of a theory-based weight-loss program for couples. Health Psychol. 2020;39(2):137–46. 10.1037/hea0000808. 31789558 10.1037/hea0000808 PMC6957719

[54] Schierberl Scherr AE, McClure Brenchley KJ, Gorin AA. Examining a ripple effect: do spouses’ behavior changes predict each other’s weight loss? Journal of obesity. 2013;2013(1):297268. 24083021 10.1155/2013/297268 PMC3777131

[55] Gellert P, Ziegelmann JP, Warner LM, Schwarzer R. Physical activity intervention in older adults: does a participating partner make a difference? Eur J Ageing. 2011;8:211–9. 28798651 10.1007/s10433-011-0193-5 PMC5547339

[56] Wang ML, Pbert L, Lemon SC. Influence of family, friend and coworker social support and social undermining on weight gain prevention among adults. Obesity (Silver Spring). 2014;22(9):1973–80. 10.1002/oby.20814. 24942930 10.1002/oby.20814 PMC4435839

[57] Rotman SA, Fowler LA, Ray MK, et al. Family encouragement of healthy eating predicts child dietary intake and weight loss in family-based behavioral weight-loss treatment. Child Obes. 2020;16(3):218–25. 10.1089/chi.2019.0119. 31829732 10.1089/chi.2019.0119 PMC7099423

[58] Sorkin DH, Mavandadi S, Rook KS, et al. Dyadic collaboration in shared health behavior change: the effects of a randomized trial to test a lifestyle intervention for high-risk Latinas. Health Psychol. 2014;33(6):566. 24884910 10.1037/hea0000063

[59] Rieger E, Lee YF, Monaghan C, Zwickert K, Murray K. Measuring social processes regarding eating, physical activity, and weight in higher-weight people: the weight-related interactions scale (WRIS). Eating Weight Disord – Stud Anorexia, Bulimia Obes. 2022;27(2):737–49. 10.1007/s40519-021-01208-2. 10.1007/s40519-021-01208-2 34041685

[60] Park Y, Dodd KW, Kipnis V, et al. Comparison of self-reported dietary intakes from the Automated Self-Administered 24-h recall, 4-d food records, and food-frequency questionnaires against recovery biomarkers. Am J Clin Nutr. 2018;107(1):80–93. 29381789 10.1093/ajcn/nqx002 PMC5972568

[61] Gorin AA, Lenz EM, Cornelius T, Huedo-Medina T, Wojtanowski AC, Foster GD. Randomized controlled trial examining the ripple effect of a nationally available weight management program on untreated spouses. Obesity. 2018;26(3):499–504. 29388385 10.1002/oby.22098 PMC5838794

